# Establishment of a molecular cytogenetic analysis for native tumor tissue of meningiomas-suitable for clinical application

**DOI:** 10.1186/1755-8166-7-12

**Published:** 2014-02-05

**Authors:** Cornelia Lerner, Ralf Ketter, Stefan Linsler, Wolfram Henn, Joachim Oertel, Steffi Urbschat

**Affiliations:** 1 Department of Otolaryngology, Saarland University, Homburg/Saar, Germany; 2 Department of Neurosurgery, Saarland University, Homburg/Saar D-66421, Germany; 3 Institute of Human Genetics, Saarland University, Homburg/Saar, Germany

**Keywords:** Meningioma, Chromosomes, Cell culture, Fluorescence *in situ* hybridization

## Abstract

**Background:**

Meningiomas are mostly benign tumors which arise from the meninges. They are among the cytogenetically best-studied solid tumors, mostly displaying a normal karyotype or, as a typical primary aberration, monosomy of chromosome 22. Further secondary chromosomal aberrations, especially the deletion of chromosome 1p, are correlated with increasing biological aggressiveness up to malignancy. These data are derived from the cytogenetical characterization of 661 meningiomas, from which the genetic progression score (GPS) has been developed. Due to the high expenditure of time and the expert knowledge for the cytogenetical characterization, the aim of this work was to establish an equally reliable yet more rapid clinical diagnosis based on fluorescence *in situ* hybridization (FISH) on meningiomas. Thus a comparison between the native tumor tissue and the primary culture of the same tumor was done in order to determine the most efficient method for a molecular cytogenetic characterization. The diagnostic procedure has to deliver fast and robust results, since they must enable the attending physician to plan the appropriate follow-up regimens for the patients. All in all, preparations of native tumor tissue as well as preparations of cell culture of 22 meningiomas were tested with FISH for aberrations concerning the prognostically relevant chromosome regions 1p and 9p, and the chromosomes 10, 14, 18 and 22 in comparison with the particular karyotypes revealed by conventional karyotyping using G-banding.

**Results:**

The FISH examinations between native and cultured cells showed an accordance of 93.4%. The comparison of FISH data and karyotyping presented accordance to the greatest possible extent concerning the chromosomes 14, 18 and 22, but to detect the progression associated losses of 1p and 9p FISH is the most sensitive method.

**Conclusions:**

The raised data reveal that both methods can be used for a significant analysis of chromosome aberrations on meningiomas. As a result of that the complex primary culture could also be avoided. Therefore a clinical diagnosis based on FISH on meningiomas is at hand for the assignment of patients to a suitable follow-up regimen.

## Background

Meningiomas are typically benign and slow-growing tumors arising from arachnoidal cells of the leptomeninges of brain and spinal cord. They belong to the cytogenetically best-studied solid tumors with a normal karyotype or, typically, monosomy of chromosome 22, which was first mentioned by Zang and Singer in 1967 [1]. The loss of chromosome 22 [1-3] is followed by clinically relevant secondary losses of complete chromosomes or parts of them. The chromosomes 6, 10, 14, 18 and 19 and partial or complete loss of the short arm of one chromosome 1 or 9 are particularly affected [3-20], whereby increasing hypodiploidy is strongly correlated with increasing malignancy. According to a study of 661 meningiomas [11], more than 75% of meningiomas belong to the common type (WHO grade I), ~20% belong to the atypical or intermediate type (WHO grade II) and only ~3% belong to the anaplastic type (WHO grade III). Approximately 5% of all meningiomas, consisting of all anaplastic meningiomas and a minority of the other subtypes, show an aggressive clinical behaviour with increased risk of tumor recurrence. However, even low-grade meningiomas exhibit an unexpectedly high recurrence rate [21-33]. To recognize the patients with the high risk of tumor recurrence, Ketter *et al.*[8] developed a neurosurgeon’s postoperative management protocol, which is based on the cytogenetical data of 661 meningiomas. The raised data of these cytogenetically characterized meningioma patients, including 53 patients with tumor recurrence, enable an application of a new mathematical model, in which the cancer development is described biomathematically by mixtures of oncogenetic tree models. The mixture model proposed by Ketter *et al.*[9] allows every genetic pattern of a meningioma to be explained, and the probabilistic framework facilitates for converting probalilities to times and thus assigning a genetic progression score (GPS) to every tumor sample. So the GPS allows a better assessment of the prognosis of meningiomas than traditional categorical cytogenetic markers and provides a further relevant discrimination of high risk and low risk groups within the same WHO grade. To plan the appropriate follow-up regimens for the patients, clinical diagnostics have to deliver fast and significant results.

The aim of this work was to establish a clinical diagnostic procedure, based on fluorescence *in situ* hybridization (FISH) on meningiomas to determine the genetic pattern for calculating the GPS. A comparison between the native tumor tissue and the primary culture of the same tumor was done in order to determine the most efficient method for a molecular cytogenetic evaluation.

## Results

### Primary tumor cells

Primary cultures plus native tissue samples from 22 meningiomas were established. To calculate the growing period of the primary culture of meningiomas, the period between the establishment and the first splitting of the primary culture was determined. The average growing time was 17.95 days with the shortest time of 7 days and the longest time of 38 days (Table 1). It should be noted, however, that the normal range of all primary cultures is between 7 days and 25 days. Three meningiomas fell out of this range, because they showed growing periods of 31 days, 32 days and 38 days. In addition, none of these three meningiomas exhibited the typical monosomy 22. Therefore we had to assume that the primary cultures with a growing time of about four weeks showed no tumor cells. Probably the tumor samples ontained no viable tumor cells.

**Table 1:** Comparison of the chromosomal aberrations detected by fluorescence in situ hybridization in native tumor tissue (dapped slides), and in vitro cell culture with classic cytogenetic findings

**Case**	**Primary cell culture growing period [Days]**	**FISH dapped slides**						**FISH cell culture**						**Cytogenetic**						
		**1p**	**22**	**9p**	**10**	**14**	**18**	**1p**	**22**	**9p**	**10**	**14**	**18**	**1p**	**22**	**9p**	**10**	**14**	**18**	**Additional aberrations**
T6801	15	1	1	0	-	0	1	1	1	0	-	1	1	0	1	0	0	0	1	0
T6805	7	0	1	0	-	0	0	0	1	0	-	0	0	0	1	0	0	1/2	0	1
T6815	13	1	1	0	-	1	0	0	0	0	-	1	0	0	1	0	0	0	0	0
T6821	11	0	0	0	-	0	0	0	0	0	-	0	0	0	0	0	0	0	0	0
T6849	18	0	0	0	-	0	0	0	0	0	-	0	0	-	-	-	-	-	-	-
T6852	25	1	1	0	0	0	0	1	1	0	0	0	0	0	0	0	0	0	0	0
T6855	32	0	0	0	0	0	0	0	0	0	0	0	0	0	0	0	0	0	0	0
T6856	17	0	0	0	0	1	1	0	0	0	0	0	0	-	-	-	-	-	-	-
T6857	13	0	1	0	0	0	0	0	1	0	0	0	0	0	1	0	1	1	1	1
T6858	38	0	0	0	0	0	0	0	0	0	0	0	0	0	0	0	0	0	0	0
T6860	21	-	-	0	0	0	0	0	0	0	0	0	0	0	0	0	0	0	0	1
T6861	21	0	1	0	0	0	0	0	1	0	0	0	0	0	1	0	1	0	0	1
T6863	15	0	1	0	0	0	0	0	1	0	0	0	0	0	1	0	0	0	0	0
T6886	18	1	1	-	-	-	-	1	1	0	0	0	0	0	1	0	0	0	0	1
T6889	31	0	0	0	0	0	0	0	0	0	0	0	0	0	0	0	0	0	0	0
T6894	14	0	0	0	0	0	0	0	0	0	0	0	0	0	0	0	0	0	0	0
T6922	9	0	0	1	0	0	0	0	0	1	0	0	0	-	-	-	-	-	-	-
T6926	13	0	1	0	0	0	0	0	1	0	0	0	0	0	0	0	0	0	0	1
T6927	15	0	1	2	2	2	0	0	1	0	0	0	0	0	0	0	0	0	0	0
T6930	18	1	0	0	0	0	0	1	0	0	0	0	0	-	-	-	-	-	-	-
T6934	11	0	0	0	0	0	0	0	0	0	0	0	0	0	0	0	0	0	0	0
T6940	20	0	0	0	0	0	0	0	0	0	0	0	0	0	0	0	0	0	0	0
17,95		5	10	2	1	3	2	4	9	1	0	2	1	0	7	0	2	2	2	

0: normal/ 1: loss/ 2: gain/ - no results.

### Conventional karyotyping using G-banding

**Figure 1:**
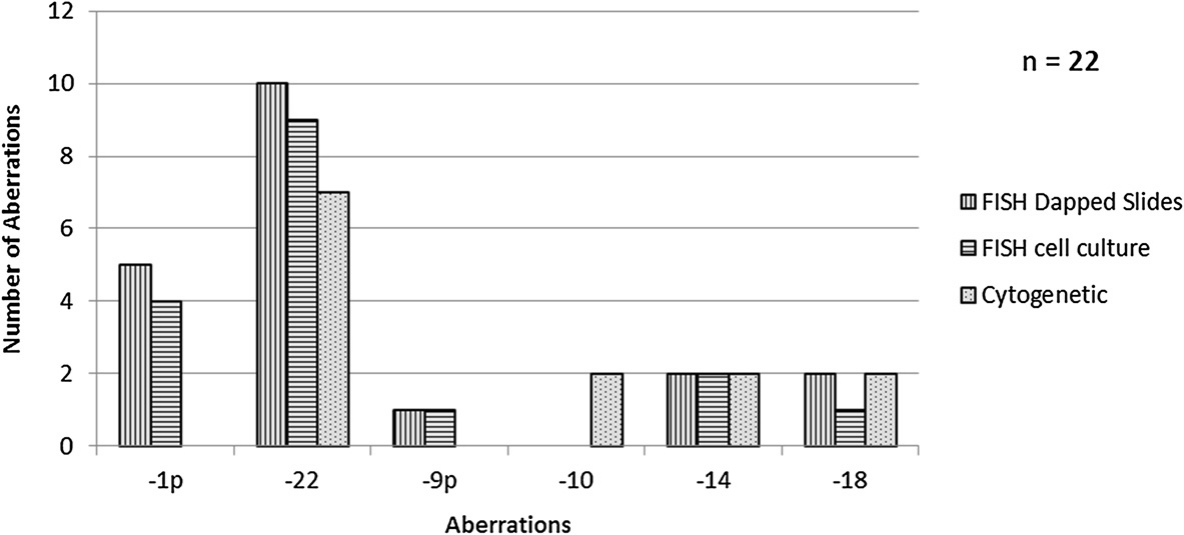
**Number of chromosomal aberrations in primary cell culture versus native tumor tissue detected using fluorescence *in situ *** **hybridization and karyotyping (22 cases analyzed).**

Microscopic karyotyping showed in a total of nine cases (40.9%) no numerical or structural aberrations. Two further cases (9.1%) showed no numerical or structural aberrations regarding the chromosomes 1, 9, 10, 14, 18 and 22, but losses of chromosome 3, 4 and X were detected as atypical anomalies. In two cases (9.1%), the monosomy 22 was the only aberration, and in one case (4.5%), monosomy 18 in addition to monosomy 22 was detected. Furthermore, (13.6%) the monosomy 22 was accompanied by other secondary losses like losses of chromosome 10, 14, 18 and further atypical chromosome aberrations in three cases. One further case (4.5%) exhibited a complex karyotype containing typical monosomy 22. In four cases (18.2%), a chromosome preparation was not possible. In any karyotyped cases aberrations regarding the chromosome regions 1p and 9p were not found. In summary the most frequent aberration was monosomy 22 followed by loss of chromosome 10, 14 and 18 which was observed in 2 cases each (Table 1, Figure 1).

### Fluorescence in situ hybridization of the tumor cells

In this study, the examination with FISH in the native tumor tissue (Figure 1; Table 1) showed in a total of nine cases (40.9%) no numerical or structural aberrations regarding the chromosomal regions 1p36, 9p21, 10q23, 14q24, 18q21 and 22q11. In five cases (22.7%), the monosomy 22 was the only aberration and in further five cases (22.7%), the monosomy 22 was accompanied by other secondary losses. Three meningiomas (13.6%) showed no aberration concerning chromosome 22, but a loss of chromosome 1p and 9p in one case each and a loss of 14 and 18 in the third case.

**Figure 2:**
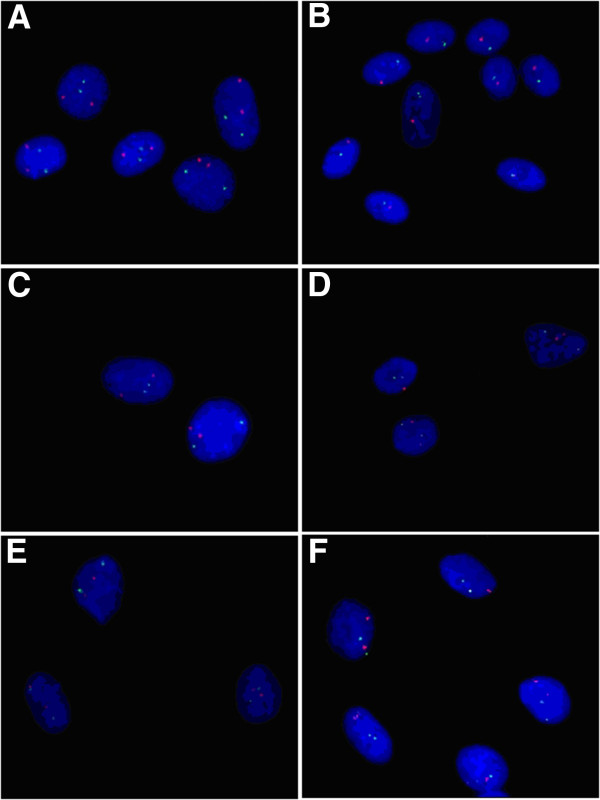
**Representative pictures of meningioma primary culture cells using fluorescence *in situ *hybridization with probes (A/B: CL 1p36/22q11, C/D: CL 9p21/10q23, E/F: CL 14q24/18q21) from MetaSystems GmbH. A/C/E**: normal cells. **B**: monosomy 22 in comparison with loss of 1p. **D**: loss of 9p. **F**: monosomy 14.

The cell culture presented in a total of ten cases (45.5%) no numerical or structural aberrations regarding the chromosomal regions 1p36, 9p21, 10q23, 14q24, 18q21 and 22q11 using FISH analysis. In six cases (27.3%), the monosomy 22 was the only aberration, and in further three cases (13.6%) the monosomy 22 was accompanied by other secondary losses. Three meningiomas (13.6%) showed no aberration concerning chromosome 22, but a loss of chromosome 1, 9 and 14 in one case each (Figures 1 and 2; Table 1).

In a total of five cases (22.7%), the evaluation of chromosome 10 was not possible concerning the native tumor tissue as well as the cell culture because of technical problems. Additionally, in one case, the evaluation of the chromosomes 1 and 22 and, in one further case, the evaluation of the chromosomes 9, 10, 14 and 18 was not possible in the native tumor tissue because of strong autofluorescence (Table 1).

In summary, the most frequent detected aberration using FISH was monosomy 22 followed by loss of chromosome region 1p and monosomy 14 in the native tumor tissue as well as in the cell culture.

### Comparison of karyotyping and FISH data

The comparison of karyotyping and FISH data presented accordance to the greatest extent concerning the chromosomes 14, 18 and 22. The losses of chromosome regions 1p and 9p were not detectable using conventional karyotyping and the FISH showed no losses of chromosome 10 (Figure 1).

### Comparison of FISH data from native and cultured tumor cells

**Figure 3:**
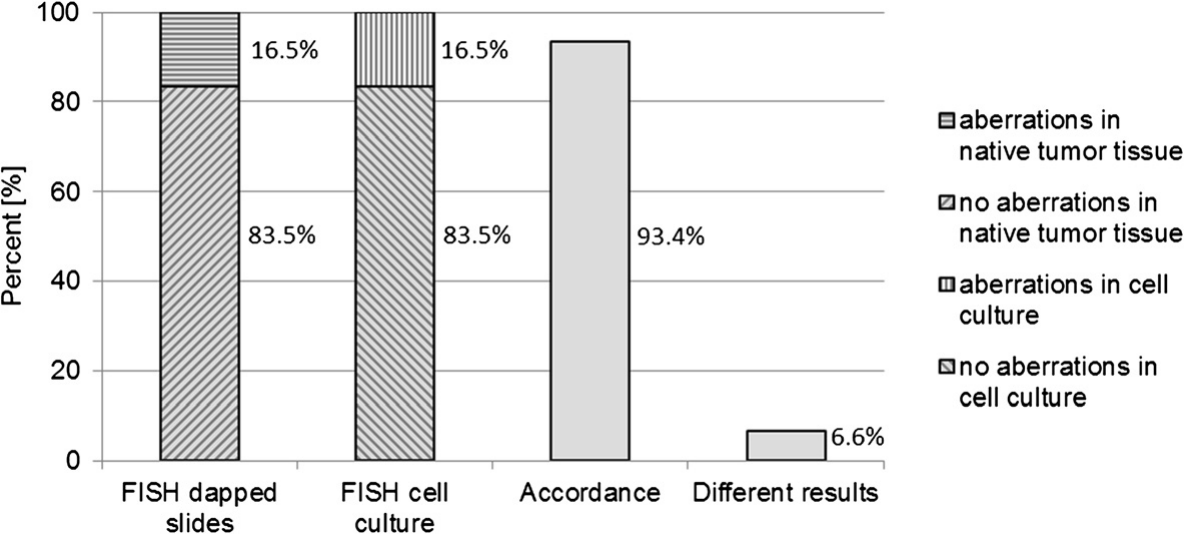
**Comparison of chromosomal aberrations between native tumor tissue and primary cell culture using fluorescence *in situ *** **hybridization.**

The comparison of FISH data from native and cultured tumor cells showed an accordance of 93.4% (Figure 3). Moreover, both native tumor tissue and cell culture present a total of 83.5% inconspicuous karyotype and 16.5% aberrations. In a total of four FISH examinations (3.3%), chromosomal aberrations were found in native tumor tissue but not in the primary tumor cell culture. Conversely, there were chromosomal aberrations in the primary tumor cell culture which was not shown in the native tumor tissue in four other examinations (3.3%). An explanation for the changes is the use of different native tumor tissue fragments.

## Discussion

### Clinical and genetic background

Most of the meningiomas classified as WHO grade I behave in a benign fashion as predicted by their histology: patients who are treated by complete surgical resection are usually cured and remain free of tumor recurrence. But grade I meningiomas may also present with grossly invasive tumor recurrence [11,34]. On the other hand, a complete surgical removal, the most important factor in preventing recurrence, is sometimes hard to achieve, as severe neurological impairment must be avoided, and even some completely resected tumors recur.

Actually, a main objective of meningioma research is to detect these meningiomas and to find predictive marker for recurrence. Many approaches to the subject have been reported. The most reliable marker is loss of chromosome 22 and the most promising prognostic marker is loss of chromosome region 1p. Recent studies have shown that meningiomas are cytogenetically heterogeneous tumors which frequently display complex karyotypes (more than three numerical or structural aberrations) [3-20]. Therefore a better treatment and advice of meningioma patients after neurosurgical procedures may be possible using a fast and reliable genetic diagnostic tool. Since now, there is no established method to detect typical genetic findings in meningiomas in the daily routine for the surgeon which would provide a fast and valid result.

### Karyotyping and FISH

In the presented study, we analysed 22 meningiomas by conventional karyotyping and FISH analysis. Both methods were compared and evaluated. The examination with FISH showed - as expected based of recent reports [1,2,35-37] - monosomy 22 as the most frequent aberration. The second most frequent aberration was the loss of the chromosome region 1p followed by the loss of chromosome 14. Furthermore there were numerical and structural aberrations of the chromosomes 9, 10 and 18 in some cases. Our results showed much more chromosomal losses than gains in comparison to a study of Sayagués and colleagues [16]. Chromosomal gains were detected in the native tumor tissue using FISH in one case (4.5%) but not in cell culture. Using conventional karyotyping the cell culture presented gains of chromosomes in three cases (13.6%). The other aberrations in the cell culture were chromosomal losses.

The karyotyping and FISH data show accordance to the greatest possible extent concerning the chromosomes 14, 18 and 22. The losses of chromosome regions 1p and 9p, which were detected using FISH, were not observed using karyotyping. One possible reason is the evaluation of 200 cell nuclei for FISH analysis in comparison to maximal 10 metaphases for karyotyping. For such evidence the FISH analysis is the most sensitive method. Particularly in view of progression associated marker the losses of 1p and 9p are important for the clinical application to recognize the patients with the high risk of tumor recurrence.

Generally the loss of chromosome 10 is a typical but rare event in meningiomas. In the present study no statement is possible because of the too small sample number. Further analysis is necessary.

Due to unsuitable tissue or insufficient fixation, in 4.5% of the examinations of native tumor tissues, FISH was not successful and the evaluation of 200 cell nuclei required for statistical analysis was not always ensured. Furthermore the autofluorescence complicated the analysis and extended the time to count the signals in the cell nuclei.

There was a chromosomal aberration in the native tumor tissue which was not detected in the cell culture in a total of four FISH examinations. Conversely, in four other examinations, there were chromosomal aberrations in the cell culture which were not shown in the native tumor tissue. Thereby we should notice that even if the FISH analysis is a well established method nevertheless an agreement of 100% cannot be achieved because of the use of heterogeneous biological material and different preparation techniques. The advantages of the native tumor tissue preparations were the use of native biological material and the avoidance of time-consuming and expensive cell culture preparation. The disadvantage was that the preparation quality was not as good as the cell culture preparation. The advantages of the cell culture preparations were the isolated cell nuclei on the object slides, the particularly clear FISH signals, and the easy and the fast analysis of 200 cell nuclei. The disadvantages of the cell culture preparations were the time-consuming primary culture and the possibility that the primary culture may show no growth.

### Clinical application

The raised data reveal that both methods can be used for a valid analysis of chromosome aberrations on meningiomas and allows the following proposal: For the clinical diagnosis, native tumor tissue preparations will be prepared, and the FISH investigations can be performed immediately. In laboratories with special equipment for cell culture, primary tumor cells can be cultured additionally. The cultivation takes place until the primary culture shows a closed cell layer. This takes 17.95 days in average, according to our experience. Then the cells will be dispersed and cell culture preparations will be made. After three days the FISH can take place. If there is no primary cell culture available, the FISH analysis of the native tumor tissue preparations is sufficient due to the presented data. Furthermore the possibility consist to isolate DNA, RNA or proteins from the primary culture or to perform immunostainings. Thus a reliable diagnostic tool is nearly guaranteed for the patients.

The development of an easy and credible method for chromosomal analysis in meningiomas is important for the translational import in clinical routine. Only if we are able to establish a method into the clinical routine without much more effort, we will have a benefit for the patients in the future. In the presented work, we presented for the first time a reliable and smart method to analyse the prognostically important chromosomal aberrations in meninigomas. The introduction of the FISH diagnostics in meningiomas will influence the postoperative management of this patient collective in future.

## Conclusions

In conclusion, the analysis of chromosomal aberrations in meningiomas based on FISH delivers fast and significant results. The study demonstrates the high sensitivity and specificity of FISH for detecting chromosomal and genetic abnormalities specific to meningioma cell cultures and tissue.

**Figure 4:**
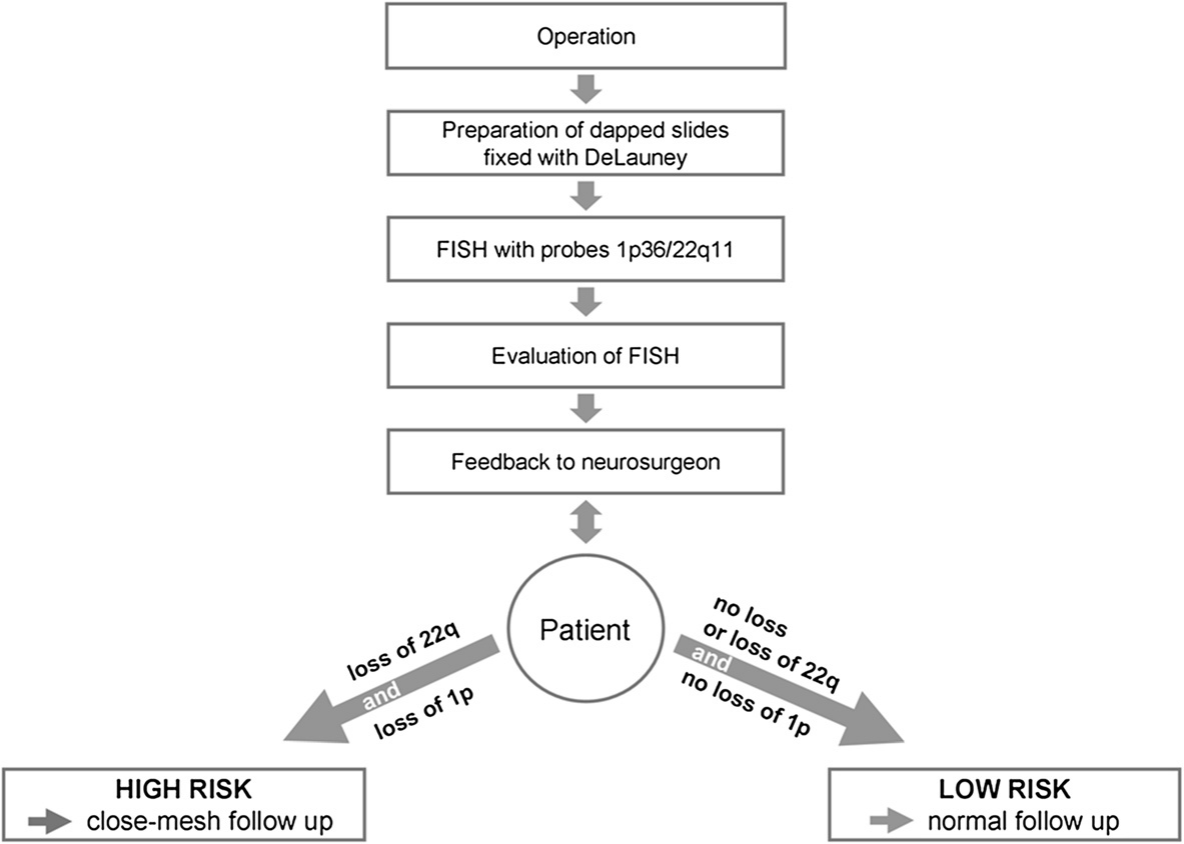
The workflow of the cytogenetic diagnostic in clinical practice.

A recommendation for routine use of FISH based analysis is displayed in Figure 4. During the operation the dapped slides should be prepared, and the FISH investigations can be performed in the laboratory immediately. The use of probes 1p36 and 22q11 is sufficient because loss of chromosome 22 is a typical general marker for meningiomas, and loss of chromosome region 1p is the most distinctive prognostic marker. According to the result of FISH analysis, the laboratory informs the neurosurgeon about the risk level of meningioma, which allows for assignement of the patients to a suitable follow-up schedule. Meningioma patients with loss of chromosome region 1p will receive close-mesh follow up and will receive early treatment if the tumor recurs.

FISH is therefore a useful adjunct to histopathology for analyzing fresh tissue or cell culture. The results of the FISH analysis allow a classification of the patients into a suitable follow-up and an early treatment of high risk meningiomas. The results of the FISH analysis are as reliable as other cytogenetic methods for clinical routine, but quicker and cheaper. In the future, the FISH analysis should be tested in a large cohort of meningioma patients to validate and establish this molecular biological tool.

## Methods

### Patient population

A study on 22 meningiomas of patients [male 4/female 18] operated at the Department of Neurosurgery, Saarland University was performed. The average patient age was 57.7 years. The 22 meningiomas comprised 16 tumors of WHO grade I, 5 tumors of WHO grade II and 1 tumor of WHO grade III (Table 2).

**Table 2:** Correlation between clinical variables and WHO tumor grade in meningiomas

	**Total**	**WHO grade I**	**WHO grade II**	**WHO grade III**
Number of patients (%)	22	16 (72,73)	5 (22,73)	1 (4,54)
Age in years	Ø 57,7	Ø 59,4	Ø 56,2	Ø 38,0
Gender (females/males)	18/4	13/3	5/0	0/1
Localisation (%)				
Convexity	12 (54,5)	8 (36,4)	3 (13,6)	1 (4,5)
Skull base	10 (45,5)	8 (36,4)	2 (9,1)	0 (0)
Spinal	0 (0)	0 (0)	0 (0)	0 (0)

### Cell culture and preparation

From each meningioma, fragments of the tumor were placed in Dulbecco’s Modified Eagle Medium (GIBCO®) with 1% penicillin/streptomycin immediately after surgery. In the neurooncological laboratory one tumor fragment was used for a primary culture. For that, the tumor sample was minced with a scalpel and small scissors. The cells were suspended in Dulbecco’s Modified Eagle Medium containing 10% fetal calf serum, 1% non-essential amino acids and 1% penicillin/streptomycin, and were distributed into 25 cm^3^ cell culture flasks. The incubation of the primary cultures took place at 37°C with 5% CO_2_ in air, whereby the medium was changed twice per week. The chromosome preparations and Giemsa banding were performed according to standard procedures [38]. The Software IKAROS (Metasystems GmbH, Altlussheim, Germany) was used to generate the karyotypes.

For FISH analysis the primary tumor cells were dispersed with 0.05% Trypsin-EDTA (GIBCO®). The dispersed cells were suspended by centrifugation at 1000 rpm for 8 minutes. After this, the supernatant was discarded and the pellet was treated with 0.075 M KCl solution. The cell suspension was centrifuged at 1000 rpm for 8 minutes. The supernatant was once again discarded and the cells were fixed with fixative (methanol/acetic acid, 3:1) for one hour. After fixation the cell suspension was dropped onto object slides.

### Preparation of dapped slides

In addition to cell culture, one tumor fragment was used for the preparation of dapped slides. Therefore a fragment of the tumor was dabbed onto object slides coated with silane and was fixed with DeLauney fixative (acetone/ethanol, 1:1, with 0.05% trichloroacetic acid). The storage was made at −20°C.

### Fluorescence in situ hybridization

Tissue specimens from tumors were obtained freshly after surgery and dabbed on microscope slides for FISH analysis. The slides were treated with RNase A for thirty minutes at 37°C and placed three times in 2xSSC for five minutes at room temperature (RT). Cells were digested in 100 ml 0.01 M HCl with 10 mg pepsin (Serva) 0,7 mA for 1 min and 45 sec at 37°C. Slides were dipped in 1xPBS for 5 min, 4%PFA/1×PBS for 10 min for fixation and 1xPBS for 5 min. They were then dehydrated in 70%, 80%, 95% ethanol and air-dried. Dual-probe hybridization was performed using locus-specific probes for 1p36, 9p21, 10q23, 14q24, 18q21 and 22q11 which were generated by Metasystems GmbH (Altlussheim, Germany). Then probes were pipetted on slides and denatured for 2 minutes at 75°C with the target. Afterwards, they were incubated overnight at 37°C in a humidified chamber. Stringency washes were performed in 0.4×SSC for 2 minutes at 72°C and 2×SSC/0.05% Tween-20 for 30 sec at RT. Finally, slides were counterstained with DAPI antifade (Vectashield, Vector Laboratories).

At least 200 non-overlapping nuclei per sample were counted for evaluation according to the Hopman criteria [39], using an Olympus AX70 fluorescence microscope. Cut-offs for alterations were determined by comparison with normal human lymphocytes as control samples at 10% for deletions of 1p36, 9p21, 10q23, 14q24, 18q21 and 22q11.

### Ethical approval

Written informed consent was obtained from each patient participating in the study. We have a positive vote of the Ethics committee of the Saarland University (Ethik-Nr. 178-07).

## Abbreviations

FISH: Fluorescence *in situ* Hybridisation; GPS: Genetic progression score; WHO: World Health Organization.

## Competing interests

The authors declare that they have no competing interests.

## Authors’ contributions

CL carried out the cultivation of the primary tumor cells, the production of the cultured primary cell preparations, and the fluorescence *in situ* hybridization with their analysis and drafted the manuscript. RK operated the patients, collected the tumor material, participated in the design of the study and revised the manuscript critically for important content. SL made the native tumor tissue preparation after operation, participated in the fluorescence *in situ* hybridization analysis and helped to draft the manuscript. WH participated in the design of the study and revised the manuscript critically for important content. JO operated the patients, collected the tumor material and gave final approval of the version to be published. SU conceived of the study, and participated in its design and coordination and involved in drafting the manuscript. All authors read and approved the final manuscript.
